# Dyad Arrangement Affects Perceived Valence Intensity

**DOI:** 10.1007/s42761-025-00312-1

**Published:** 2025-06-25

**Authors:** Mahsa Barzy, Richard Cook, Katie L. H. Gray

**Affiliations:** 1 School of Psychology and Clinical Language Sciences, University of Reading, Reading, England U.K.; 2 School of Psychology, University of Kent, Kent, U.K.; 3 School of Psychology, University of Leeds, Leeds, U.K.

**Keywords:** Social Interaction Perception, Social Perception, Valence, Intensity

## Abstract

The perception of emotional expressions is affected by our knowledge and experience, and the context in which they are presented. Social interactions are a natural context in which expressions are viewed, yet we are only beginning to understand how they impact our perception. In three online studies, we investigated whether social interaction contexts impact on the perceived valence intensity of emotional whole-body stimuli. We manipulated whether the dyads were presented within a social interaction or not by changing their arrangement (face-to-face, and back-to-back, respectively). Emotionally expressive dyads were presented, where both individuals expressed the same basic emotion (happy-happy, angry-angry, or neutral–neutral), and we measured the perceived intensity of the interactants’ valence. In Experiment 1, participants (*N* = 68) perceived happy and angry bodies to be more intensely positive and negative, respectively, when presented face-to-face than back-to-back for an unlimited duration. In Experiment 2, we limited the presentation duration to 500ms, and found that participants (*N* = 65) perceived angry bodies as more intensely negative when presented face-to-face than back-to-back. In Experiment 3, we replaced one of the interactants with an arrow, and manipulated their arrangement. Participants (*N* = 64) rated the intensity of the bodies similarly irrespective of their arrangement with the arrows. Our findings show that interaction contexts influence the perception of interactants’ valence intensity, and the effects are not driven by attentional cueing. These results have implications for how interactions are perceived, which may inform how we respond when we encounter groups of people in everyday life.

Facial expressions and body postures provide us with a wealth of information in social contexts and help us to shape our social interactions (Adolphs, 2002; de Gelder, 2006; Öhman, 2002; van Kleef et al., 2016). In neurotypical observers, facial and bodily emotions are processed quickly (Batty & Taylor, 2003; Wronka & Walentowska, 2011), and guide social approach decisions (Marsh et al., 2005).

Previous research indicates that the perception of facial and bodily expressions is informed by our knowledge and experience (Feldman-Barrett et al., 2011; de Gelder et al., 2006). The perception of emotion in faces (Aviezer et al., 2012; de Gelder & Vroomen, 2000; Righart & de Gelder, 2008) and bodies (Kret & de Gelder, 2010; Reschke & Walle, 2021) is critically affected by the context in which the stimuli are viewed. For example, the same facial expression varies in appearance when presented in the context of different body postures (Aviezer et al., 2012), and there is evidence that this occurs early in visual processing (Aviezer et al., 2008; Meeren et al., 2005). Most of the research to date has presented lone individuals, but some studies have investigated the perception of emotion when more than one face is presented. For example, studies show that congruent emotion categorisation is facilitated by a context face (Mumenthaler & Sander, 2012), and increased when social appraisal is implied (Mumenthaler & Sander, 2012). These effects are observed when the context stimuli are presented briefly (30ms; Mumenthaler & Sander, 2015).

There has been a growing interest in how observers process scenes containing dyads (two individuals) that are engaged in a social interaction (Abassi & Papeo, 2020; Barzy et al., 2023; Bunce et al., 2021; Gray et al., 2017; Isik et al., 2017; Papeo et al., 2017; Quadflieg & Koldewyn, 2017; Vestner et al., 2019). It is thought that facing stimuli are perceived as a social interaction, whereas non-facing stimuli (i.e., those presented back-to-back) are not afforded this interpretation (Papeo et al., 2019; Vestner et al., 2019). One view suggests that social interactions capture attention because of their importance in the navigation of our social world (Papeo, 2020). An alternative view is that social interactions capture attention because individual bodies direct visual attention (Vestner et al., 2020).

Vestner and colleagues (Vestner et al., 2020; Vestner, Gray et al., 2021; Vestner, Over et al., 2021; Vestner, Gray et al., 2022; Vestner, Over et al., 2022) argue that manipulating the arrangement of dyads not only modulates whether the dyad is perceived as a social interaction, but also changes the spatial allocation of attention. They suggest that facing dyads enhance attention on or around the dyad, which leads to faster target detection in visual search tasks (Vestner et al., 2020), but could also drive other effects found for facing versus non-facing stimuli, such as preferential looking towards (e.g., Goupil et al., 2024), and heightened BOLD activity in response to (e.g., Abassi & Papeo, 2020) face-to-face dyads. Thus, Vestner et al. (2020) argue that where facing versus non-facing stimuli are used, it is important to examine the contribution of attentional cueing to the effect in question.

Whilst there has been an increase in research investigating how social interactions are perceived from a third-person perspective, few studies have focussed on the perception of affect in interaction contexts. In an early study, Cline (1956) noted that observers were likely to group line-drawing images of facial expressions into a single unit, with the expression of one interactant influencing the interpretation of emotion and dominance in the other. Point-light walkers have been used to investigate the categorisation of emotion in social interactions, showing it is affected by the orientation of the dyads (Clarke et al., 2005), and a temporal offset between the actors’ actions (Bachmann et al., 2022). Further, facial (Gray et al., 2017) and bodily (Abramson et al., 2021) expressions of one interactant have been found to influence the perceived expression of the other when they are presented face-to-face, but not when the same stimuli are presented back-to-back. However, none so far has attempted to examine the contribution of attentional cueing to the expression perception of dyads.

Emotion categorisation tasks are important insofar as they can tell us if the emotion itself is categorised correctly or misclassified, and which expressions are often confused with each other. Emotional intensity—where expressions can vary from being mild, to extremely intense—is another important cue that guides how we perceive and respond to individuals (Gray et al., 2020; Hess et al., 1997; Leppänen et al., 2007). The perception of emotional intensity has been found to be influenced by various factors, including facial concealment (Tsantani et al., 2022), gaze direction (Adams Jr. & Kleck, 2005), and the exaggeration of body movement (Atkinson et al., 2004). However, previous research has not examined this in relation to dyad arrangement.

Here, we investigated whether the perceived intensity of emotional dyads is affected by whether they are presented in a social interaction arrangement. In Experiment 1, we presented participants with face-to-face and back-to-back static emotional body dyads in which both individuals depicted angry, neutral, and happy emotions. Participants were given unlimited time to view the dyad, and rated one of the individuals on both their positive and negative valence intensity. In Experiment 2, in a new sample of participants, we investigated whether the effect of dyad context on valence intensity ratings was evident when the stimuli were presented for a limited duration (500ms). In Experiment 3, we replaced one of the individuals with an arrow to investigate whether directing participants’ attention to the to-be-rated stimulus affected their intensity ratings.

We predicted that that happy bodies would be rated as more positive, and angry bodies more negative, when presented face-to-face than back-to-back. We did not predict an effect of arrangement on rated valence intensity for the neutral dyads. In line with previous research on the effects of body contexts on emotional expression categorisation, we predicted that the effects would be evident when the stimuli were presented for a limited duration. Finally, we predicted that *if* the effects were driven by attentional cues, the same results would be found for emotional bodies paired with arrow stimuli.

## Experiment 1

### Method

#### Participants

For Experiment 1, 68 participants (*M*_age_ = 41.63, *SD*_age_ = 13.17; 43 females, 25 males) were recruited from www.prolific.co. To be eligible, participants were required to be aged between 18 and 65, to have normal or corrected-to-normal vision, be based in the UK, and have English as a first language. All participants gave informed consent. Ethical clearance was granted by the local ethics committee at the University of Reading and the study was performed in accordance with the ethical standards as laid down in the 6th (2008) Declaration of Helsinki. Previous research has suggested a large effect (*f* = 0.31) of dyad arrangement on emotion perception using a sensitive psychophysical task (Gray et al., 2017). To ensure we were able to detect differences, G*Power (Faul et al., 2007) was used to estimate the sample size needed for a repeated ANOVA with a medium effect size (f = 0.176) and with power of 0.85. The suggested overall sample size was 62. We exceeded this in each experiment.

#### Stimuli

**Fig. 1:**
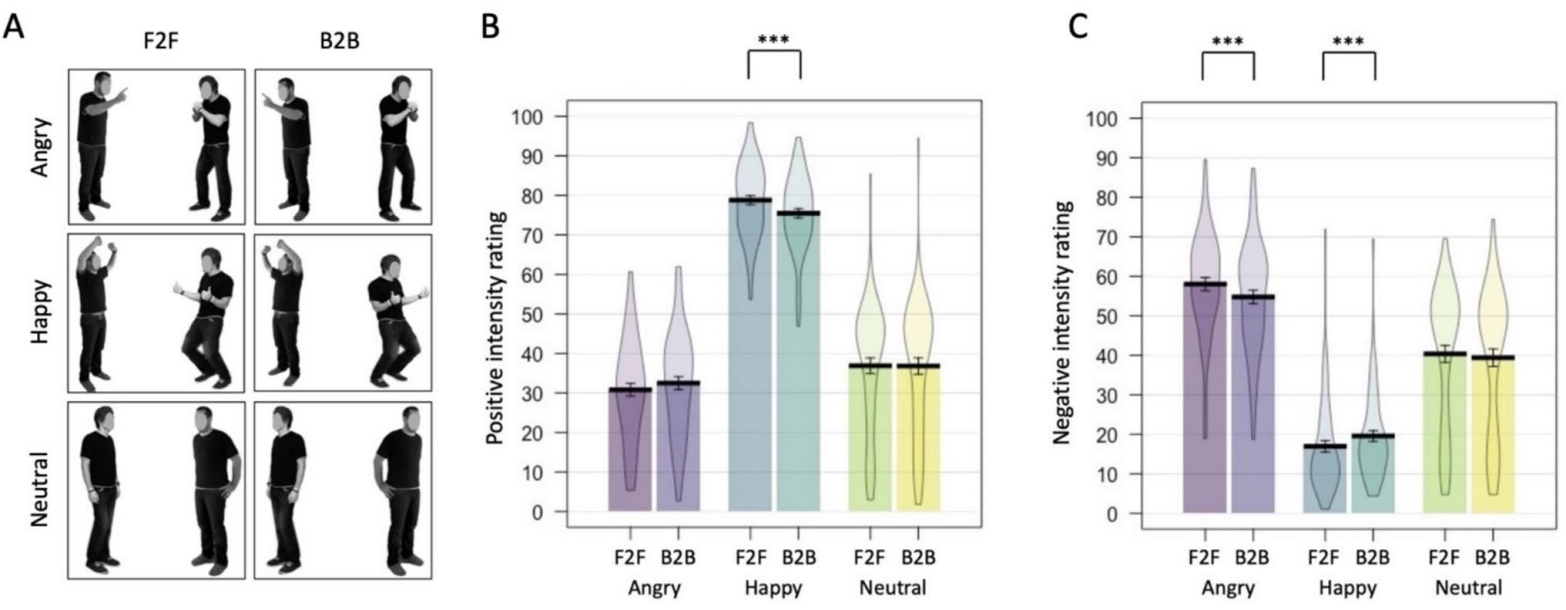
Examples of the stimuli used in Experiment 1 (A). Results from Experiment 1, for positive intensity ratings (B) and negative intensity ratings (C). Error bars = 1SEM. Significant pairwise comparisons between face-to-face (F2F) and back-to-back (B2B) dyads are indicated. *** denotes *p* < 0.001

We selected 24 models displaying standardized and validated emotional body postures from the Bochum Emotional Stimulus Set (BESST; Thoma et al., 2013), and chose images that were rotated 45° to the camera. The BESST stimuli are emotional body postures with blurred faces. We selected models from three emotional categories – neutral, happy, and angry. We arranged the models (all male) randomly into pairs of stimuli, creating 72 face-to-face dyads (24 of each emotional category; Fig. 1A). Images were first cropped, then resized, and combined into dyads in MATLAB. To create the back-to-back dyads, we flipped the facing-direction of the individuals presented in each face-to-face dyad, creating another 72 images. We also switched the side that each face-to-face stimulus was placed on (by horizontally flipping the image), before then creating another set of back-to-back images in the same way as above. Participants were shown either the first, or the second set of images (counter-balanced across participants). Therefore, each participant saw 144 dyads and 12 practice trials at the start of the experiment.

#### Design

We used a 3 Emotion (Angry, Happy, Neutral) × 2 Arrangement (Face-to-face, Back-to-back) factorial design. All experiments described were conducted online, an approach that is increasingly common. Carefully-designed online tests of cognitive and perceptual processing can yield high-quality data, indistinguishable from that collected in the lab (Crump et al., 2013; Germine et al., 2012). The experiments were conducted using Gorilla Experiment Builder, a cloud-based research platform that allows researchers to create and deploy experiments online and collect precise behavior data (Anwyl-Irvine et al., 2020). Participants were instructed to use only desktop computers or laptops.

#### Procedure

On each trial, a fixation cross was centrally presented for 1500ms, followed by the dyad. Participants were asked to “Rate the emotional intensity of the person on the < side > ”, where < side > was ‘left’ or ‘right’, and was counterbalanced across participants. After stimulus presentation, text appeared at the top of the screen reminding participants to rate the intensity of the person on the < side > . Beneath the images, two rating scales appeared, the first with the prompt “How positive?” where participants responded to the perceived positive valence (from 0 – ‘not at all positive’, to 100 – ‘extremely positive’), and the second with the prompt “How negative?” where participants responded to the perceived negative valence (from 0 – ‘not at all negative’ to 100 – ‘extremely negative’). Two scales were used to ensure the framing of the question was not driving the effects; we predicted the opposite pattern of results across the two scales (i.e., a conceptual replication). Each dyad was presented until the slider responses had been selected and participants chose to continue to the next trial by pressing the ‘Next’ button with their mouse. There were 6 blocks with 24 trials in each, and a break programmed at the end of each block. Trial-order was randomised within blocks; block-order was randomised across the experiment.

Catch trials consisted of an image of a chair. Participants were instructed to ‘rate it as 0 on the scale for both positive and negative emotion’. There was 1 catch trial in the practice trials, and 6 in the experimental blocks. Participants were required to respond to at least 5/6 correctly to be included. In total, the experiment took around 25 min.

#### Data Analysis

Intensity ratings were evaluated using both traditional null-hypothesis significance testing (NHST; α = .05, two-tailed) and Bayesian methods (JASP-Team, 2022). For NHST, where Mauchly’s test of sphericity was violated, Greenhouse-Giesser corrections were applied. For the Bayesian analyses, we used the default prior width, and interpret Bayes Factors (BF_01_) larger than 1, 3, and 10 as anecdotal, substantial, and strong evidence in favour of the null hypothesis, respectively. We interpret Bayes Factors (BF_01_) less than 1, 1/3, and 1/10 as anecdotal, substantial, and strong evidence in favour of the alternative hypothesis, respectively. Data associated with the experiments can be accessed here: https://osf.io/sz294/?view_only=eba68f3bb9334219af2d8c855737acef

## Results & Discussion

All participants responded correctly to at least 5 out of 6 catch trials; none were excluded.

### Positive Intensity Rating

The positive intensity ratings were submitted to ANOVA with Emotion (Angry, Happy, Neutral) and Arrangement (Face-to-face, Back-to-back) as within-participant variables. The analysis revealed no main effect of Arrangement [*F*(1, 67) = 2.478, *p* = .120, η_p_^2^ = 0.036], but a significant main effect of Emotion [*F*(2, 134) = 342.35, *p* < .001, η_p_^2^ = 0.836] which was qualified by an interaction between Emotion and Arrangement [*F*(1.731, 115.962) = 22.296, *p* < .001, η_p_^2^ = 0.250] (Fig. 1B).

Individuals presented in Happy face-to-face (*M* = 78.79, *SD* = 9.43) pairs were rated as significantly more positive than those in Happy back-to-back (*M* = 75.47, *SD* = 9.77) pairs [*t*(67) = 6.727, *p* < .001, *d* = 0.816, BF_01_ < 0.001]. When Bonferroni corrected, there was no significant difference between those presented in Angry face-to-face (*M* = 30.81, *SD* = 13.28) and Angry back-to-back (*M* = 32.48, *SD* = 13.52) pairs [*t*(67) = 2.357, *p* = .021, *d* = 0.286, BF_01_ = 0.575], with the Bayes Factor suggesting anecdotal support for the alternative hypothesis. There was also no significant difference between individuals presented within Neutral face-to-face (*M* = 36.88, *SD* = 16.30) and Neutral back-to-back (*M* = 36.81, *SD* = 17.09) pairs [*t*(67) = 0.145, *p* = .885, *d* = 0.018, BF_01_ = 7.435], with the Bayes Factor suggesting strong support for the null hypothesis.

There was a significant effect of Emotion in both the Face-to-face [*F*(2, 134) = 349.031, *p* < .001, η_p_^2^ = 0.839], and the Back-to-back condition [*F*(2, 134) = 309.558, *p* < .001, η_p_^2^ = 0.822], with the same pattern of effects found in both. Happy pairs were perceived as significantly more positive than those presented in Angry pairs (Face-to-face: [*t*(67) = 24.200, *p* < .001, *d* = 2.935, BF_01_ < 0.001]; Back-to-back: [*t*(67) = 23.654, *p* < .001, *d* = 2.868, BF_01_ < 0.001]), and Neutral pairs (Face-to-face: [*t*(67) = 20.317, *p* < .001, *d* = 2.464, BF_01_ < .001]; Back-to-back: [*t*(67) = 18.398, *p* < .001, *d* = 2.231, BF_01_ < 0.001]). Individuals presented within Neutral pairs were perceived as more positive than those presented within Angry pairs (Face-to-face: [*t*(67) = 3.224, *p* = .002, *d* = 0.391, BF_01_ = 0.071]; Back-to-back: [*t*(67) = 2.443, *p* = .017, *d* = 0.296, BF_01_ = 0.478] with the Bayes Factors suggesting substantial and anecdotal support for the alternative hypothesis, respectively).

### Negative Intensity Rating

The negative intensity ratings were submitted to ANOVA with Emotion (Angry, Happy, Neutral) and Arrangement (Face-to-face, Back-to-back) as within-participant variables. The analysis revealed no main effect of Arrangement [*F*(1, 67) = 2.110, *p* = .151, η_p_^2^ = 0.031], but a significant main effect of Emotion [*F*(2, 134) = 161.371, *p* < .001, η_p_^2^ = 0.707], which was qualified by an Arrangement by Emotion interaction [*F*(1.526, 102.219) = 21.433, *p* < .001, η_p_^2^ = 0.242] (Fig. 1C).

Individuals presented within Happy face-to-face (*M* = 16.96, *SD* = 11.99) pairs were rated as significantly less negative than those in Happy back-to-back (*M* = 19.57, *SD* = 11.33) pairs [*t*(67) = 5.349, *p* < .001, *d* = 0.649, BF_01_ < 0.001]. Individuals presented in Angry face-to-face (*M* = 58.03, *SD* = 13.57) pairs were rated as more negative than those in Angry back-to-back (*M* = 54.76, *SD* = 14.14) pairs [*t*(67) = 3.711, *p* < .001, *d* = 0.450, BF_01_ = 0.018]. When Bonferroni corrected, there was no significant difference between individuals presented in Neutral face-to-face (*M* = 40.35, *SD* = 17.80) and Neutral back-to-back (*M* = 39.39, SD = 18.30) pairs [*t*(67) = 2.061, *p* = .043, *d* = 0.250, BF_01_ = 1.033], with the Bayes Factor suggesting anecdotal support for the null hypothesis.

There was a significant effect of Emotion in both the Face-to-face [*F*(2, 134) = 171.620, *p* < .001, η_p_^2^ = 0.719], and the Back-to-back condition [*F*(2, 134) = 137.503, *p* < .001, η_p_^2^ = 0.672], with the same pattern of effects found in both. Individuals presented within Happy pairs were perceived as significantly less negative than those presented in Angry pairs (Face-to-face: [*t*(67) = 18.800, *p* < .001, *d* = 2.280, BF_01_ < 0.001]; Back-to-back: [*t*(67) = 17.706, *p* < .001, *d* = 2.147, BF_01_ < 0.001]), and Neutral pairs (Face-to-face: [*t*(67) = 10.605, *p* < .001, *d* = 1.286, BF_01_ < 0.001]; Back-to-back: [*t*(67) = 9.140, *p* < .001, *d* = 1.108, BF_01_ < 0.001]). Individuals presented within Neutral pairs were perceived as less negative than those presented within Angry pairs (Face-to-face: [*t*(67) = 7.752, *p* < .001, *d* = 0.940, BF_01_ < 0.001]; Back-to-back: [*t*(67) = 6.925, *p* < .001, *d* = 0.840, BF_01_ < 0.001]).

In Experiment 1, we found that dyad arrangement affected perceived emotional intensity. In this experiment, unlimited time was given to participants to respond to the stimuli (e.g., Abramson et al., 2021). However, using this method, participants’ responses could be the product of effortful and careful examination of the stimuli, rather than reflect early perceptual processes. To ascertain whether the effects of social interaction context on valence intensity ratings exist for stimuli that have a limited exposure duration, we next reduced the presentation duration of the stimuli to 500ms.

## Experiment 2

### Method

#### Participants

For Experiment 2, 68 participants were recruited from www.prolific.co. All inclusion/exclusion criteria and ethics information were the same as Experiment 1. Three participants were excluded based on their responses to the catch trials; the remaining participants responded correctly to at least 5 out of 6 catch trials. The final sample consisted of 65 participants (*M*_age_ = 39.57, *SD*_age_ = 12.57; 41 females, 22 males, 2 prefer not to say).

#### Stimuli & Design

All aspects of the experiment were the same as Experiment 1, aside from the images being presented for 500ms, a duration for which only one or two saccades tend to be made (Pelz & Canosa, 2001). Stimulus presentation was followed by a fixation cross for a further 200ms, before the response screen was presented.

## Results & Discussion

### Positive Intensity Rating

**Fig. 2:**
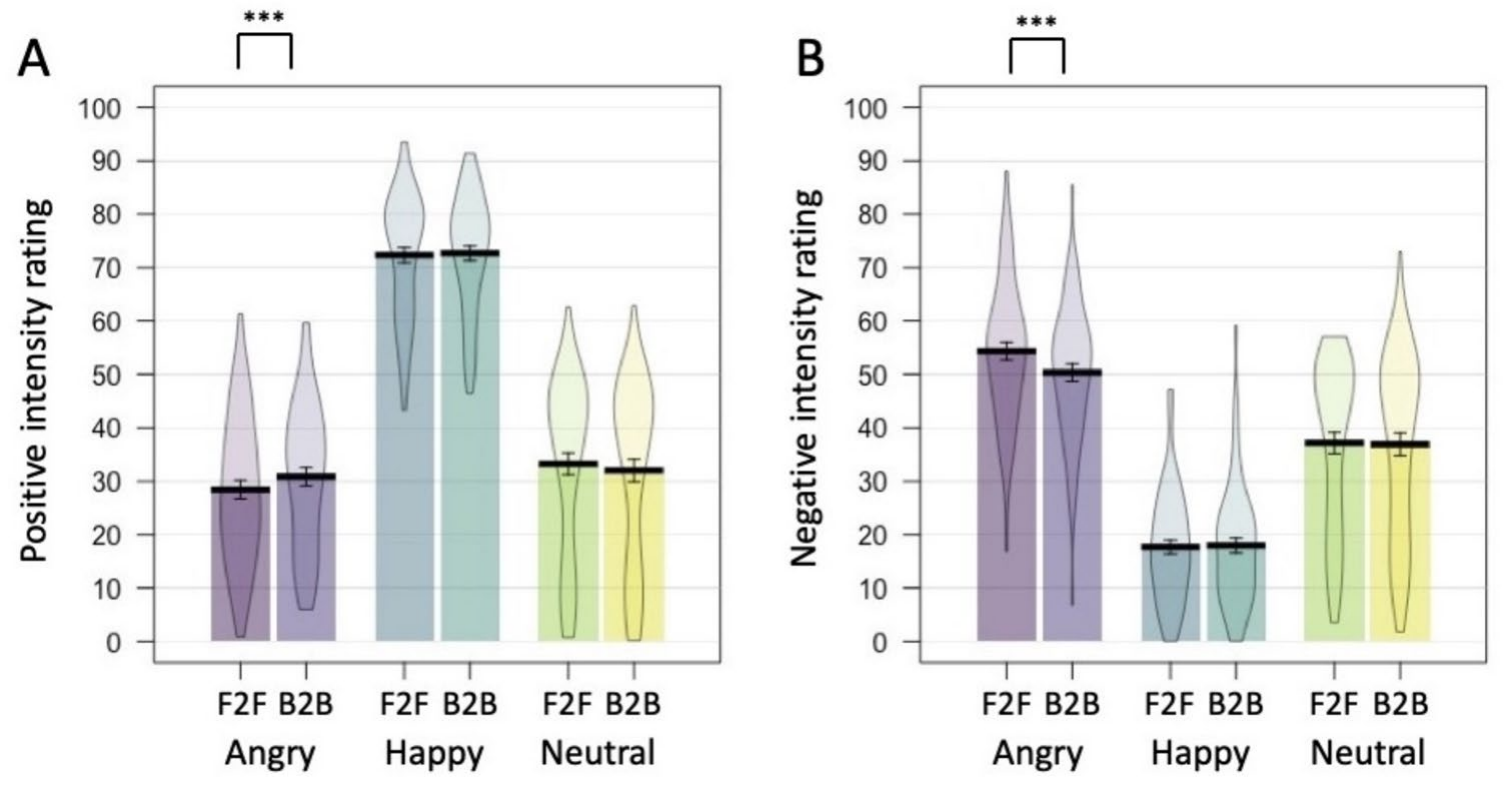
Results from Experiment 2, for positive intensity ratings (A) and negative intensity ratings (B). Error bars = 1SEM. Significant pairwise comparisons between face-to-face (F2F) and back-to-back (B2B) dyads are indicated. *** = *p* < 0.001

The positive intensity ratings were submitted to ANOVA with Emotion (Angry, Happy, Neutral) and Arrangement (Face-to-face, Back-to-back) as within-participant variables. The analysis revealed no main effect of Arrangement [*F*(1,64) = 1.503, *p* = 0.225, η_p_^2^ = 0.023], but a main effect of Emotion [*F*(1.794, 123.556) = 388.375, *p* < .001, η_p_^2^ = 0.859], was qualified by a significant Arrangement by Emotion interaction [*F*(2,128) = 10.858, *p* < .001, η_p_^2^ = 0.145] (Fig. 2A).

Individuals presented in Angry Face-to-face (*M* = 28.46, *SD* = 13.99) pairs were rated as significantly less positive than those in Angry Back-to-back (*M* = 30.88, *SD* = 13.66) pairs [*t*(64) = 3.945, *p* < .001, *d* = 0.489, BF_01_ = 0.009]. There was no significant difference between individuals presented within Happy Face-to-face (*M* = 72.31, *SD* = 11.54) and Back-to-back (*M* = 72.66, *SD* = 11.18) pairs [*t*(64) = 0.555, *p* = .581, *d* = 0.069, BF_01_ = 6.345]. Once Bonferroni corrected, there was also no significant difference between individuals presented within Neutral Face-to-face (*M* = 33.27, *SD* = 16.59) and Back-to-back (*M* = 32.03, *SD* = 17.06) pairs [*t*(64) = 2.030, *p* = .047, *d* = 0.252, BF_01_ = 1.077], where the Bayes Factor suggested anecdotal support for the null hypothesis.

There was a significant effect of Emotion in both the Face-to-face [*F*(2, 128) = 361.811, *p* < .001, η_p_^2^ = 0.850], and the Back-to-back condition [*F*(1.753, 112.174) = 377.536, *p* < .001, η_p_^2^ = 0.855], with a similar pattern of effects in both. Individuals presented within Happy pairs were perceived as significantly more positive than those presented in Angry pairs (Face-to-face: [*t*(64) = 26.178, *p* < .001, *d* = 3.247, BF_01_ < 0.001]; Back-to-back: [*t*(64) = 26.435, *p* < .001, *d* = 3.279, BF_01_ < 0.001]), and Neutral pairs (Face-to-face: [*t*(64) = 19.234, *p* < .001, *d* = 2.386, BF_01_ < 0.001]; Back-to-back: [*t*(64) = 20.005, *p* < .001, *d* = 2.481, BF_01_ < 0.001]). Individuals presented within Neutral pairs were perceived as more positive than those presented within Angry pairs when arranged Face-to-face [*t*(64) = 2.950, *p* = 0.004, *d* = 0.366, BF_01_ = 0.145], but not when arranged Back-to-back [*t*(64) = 0.747, *p* = .458, *d* = 0.093, BF_01_ = 5.627].

### Negative Intensity Rating

The negative intensity ratings were submitted to ANOVA with Emotion (Angry, Happy, Neutral) and Arrangement (Face-to-face, Back-to-back) as within-participant variables. The analysis revealed a main effect of Arrangement [*F*(1, 64) = 4.617, *p* = .035, η_p_^2^ = 0.067], and a main effect of Emotion [*F*(2, 128) = 208.490, *p* < .001, η_p_^2^ = 0.765], which were qualified by a significant Arrangement by Emotion interaction [*F*(2, 128) = 20.704, *p* < .001, η_p_^2^ = 0.244] (Fig. 2B).

Individuals presented in Angry Face-to-face (*M* = 54.32, *SD* = 13.55) pairs were rated as significantly more negative than those in Angry Back-to-back (*M* = 50.32, *SD* = 13.06) pairs [*t*(64) = 5.085, *p* < 0.001, *d* = 0.631, BF_01_ < 0.001]. There was no significant difference between individuals presented within Happy Face-to-face (*M* = 17.68, *SD* = 10.39) and Back-to-back (*M* = 17.95, *SD* = 11.36) pairs [*t*(64) = 0.320, *p* = .750, *d* = 0.040, BF_01_ = 7.000], nor between individuals presented within Neutral Face-to-face (*M* = 37.20, *SD* = 15.94) and Back-to-back (*M* = 36.95, *SD* = 17.03) pairs [*t*(64) = 0.443, *p* = .659, *d* = 0.055, BF_01_ = 6.687].

There was a significant effect of Emotion in both the Face-to-face [*F*(2, 128) = 210.802, *p* < .001, η_p_^2^ = 0.767], and the Back-to-back condition [*F*(2, 128) = 188.317, *p* < .001, η_p_^2^ = 0.746], with the same pattern of effects in both. Individuals presented within Happy pairs were perceived as significantly less negative than those presented in Angry pairs (Face-to-face: [*t*(64) = 22.841, *p* < .001, *d* = 2.833, BF_01_ < 0.001]; Back-to-back: [*t*(64) = 22.489, *p* < .001, *d* = 2.789, BF_01_ < 0.001]), and Neutral pairs (Face-to-face: [*t*(64) = 11.028, *p* < .001, *d* = 1.368, BF_01_ < 0.001]; Back-to-back: [*t*(64) = 10.447, *p* < .001, *d* = 1.296, BF_01_ < 0.001]). Individuals presented within Neutral pairs were perceived as less negative than those presented within Angry pairs (Face-to-face: [*t*(64) = 8.714, *p* < .001, *d* = 1.081, BF_01_ < 0.001]; Back-to-back: [*t*(64) = 7.655, *p* < .001, *d* = 0.949, BF_01_ < 0.001]).

In Experiment 2, dyad arrangement significantly affected perceived emotional intensity for angry bodies when the presentation duration was limited to 500ms. Next, we were interested in the effect of attentional cueing, as attention is critically related to how we process visual information (Liu et al., 2009; Pestilli & Carrasco, 2005). Recent studies have highlighted the importance of directional attention cues when investigating the perception of face-to-face versus back-to-back interactions (Vestner et al., 2020, 2021; Vestner, Gray et al., 2022; Vestner, Over et al., 2022), and the importance of including inanimate control conditions (Heyes, 2014; Santiesteban et al., 2014). In Experiment 3, we replaced one of the interactants with an arrow, which is also a robust directional cue that is hard to inhibit (Kuhn & Kingstone, 2009; Tipples, 2002). If directional cues capture attention and drive the emotional intensity effects found for facing versus non-facing dyads in Experiments 1 and 2, we expected to find the same pattern of results in Experiment 3 using human-arrow stimulus pairs.

## Experiment 3

### Method

#### Participants

For Experiment 3, 64 participants (*M*_age_ = 38.83, *SD*_age_ = 12.43; 52 females, 12 males) were recruited from www.prolific.co. All inclusion/exclusion criteria and ethics information were the same as Experiments 1 and 2.

#### Stimuli & Design

**Fig. 3:**
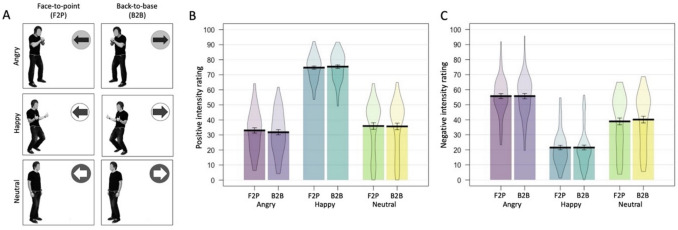
Examples of stimuli used for Experiment 3 (A), where one individual was replaced with an arrow, creating stimulus pairs which are either face-to-point (F2P) or back-to-base (B2B). Results from Experiment 3 for the positive intensity rating (B) and negative intensity rating (C). Error bars = 1SEM

All aspects of the experiment were the same as Experiment 1, apart from one of the individuals in each dyad was replaced with an arrow (Fig. 3A). Six different arrows were randomly paired with the individuals.

## Results & Discussion

All participants responded correctly to at least 5 out of 6 catch trials; no participants were excluded.

### Positive Intensity Rating

The positive intensity ratings were submitted to ANOVA with Emotion (Angry, Happy, Neutral) and Arrangement (Face-to-point, Back-to-base) as within-participant variables. The analysis revealed no main effect of Arrangement [*F*(1, 63) = 0.831, *p* = 0.365, η_p_^2^ = 0.013], but a main effect of Emotion [*F*(1.703, 107.285) = 276.247, *p* < .001, η_p_^2^ = 0.814] was qualified by a significant Arrangement by Emotion interaction [*F*(1.757, 110.709) = 4.242, *p* = .021, η_p_^2^ = 0.063] (Fig. 3B).

There was no significant difference between individuals presented within Angry Face-to-point (*M* = 33.01, *SD* = 13.59) and Back-to-base (*M* = 31.78, *SD* = 13.74) pairs [*t*(63) = 2.374, *p* = 0.021, *d* = 0.297, BF_01_ = 0.545], Happy Face-to-point (*M* = 74.76, *SD* = 8.59) and Back-to-base (*M* = 75.41, *SD* = 9.28) pairs [*t*(63) = 1.147, *p* = .256, *d* = 0.143, BF_01_ = 3.898], nor Neutral Face-to-point (*M* = 35.92, *SD* = 17.37) and Back-to-base (*M* = 35.68, *SD* = 17.50) pairs [*t*(63) = 0.778, *p* = .439, *d* = 0.097, BF_01_ = 5.459]. Bayesian statistics on the pairwise comparisons suggest strong support for the null hypothesis for happy and neutral expressions. For angry expressions, where anecdotal support for the alternative hypothesis was suggested, the effect was in the opposite direction to the effect found in Experiments 1 and 2.

There was a significant effect of Emotion in both the Face-to-point [*F*(1.722, 108.505) = 256.698, *p* < .001, η_p_^2^ = 0.803], and the Back-to-base condition [*F*(1.718, 108.205) = 282.637, *p* < .001, η_p_^2^ = 0.818], with the same pattern of effects in both. Individuals presented within Happy pairs were perceived as more positive than those presented in Angry pairs (Face-to-point: [*t*(63) = 21.926, *p* < .001, *d* = 2.741, BF_01_ < 0.001]; Back-to-base: [*t*(63) = 22.810, *p* < .001, *d* = 2.851, BF_01_ < 0.001]), and Neutral pairs (Face-to-point: [*t*(63) = 15.973, *p* < .001, *d* = 1.997, BF_01_ < 0.001]; Back-to-base: [*t*(63) = 16.572, *p* < .001, *d* = 2.071, BF_01_ < 0.001]). There was no significant difference between Angry and Neutral pairs (Face-to-point: [*t*(63) = 1.640, *p* = .106, *d* = 0.205, BF_01_ = 2.060]; Back-to-base: [*t*(63) = 2.261, *p* = .027, *d* = 0.283, BF_01_ = 0.688]), with Bayes Factors suggesting anecdotal support for the null and the alternative hypothesis in the Face-to-point and Back-to-base conditions, respectively.

### Negative Intensity Rating

The negative intensity ratings were submitted to ANOVA with Emotion (Angry, Happy, Neutral) and Arrangement (Face-to-point, Back-to-base) as within-participant variables. The analysis revealed no main effect of Arrangement [*F*(1, 63) = 1.372, *p* = .246, η_p_^2^ = 0.021], a significant main effect of Emotion [*F*(2, 126) = 161.412, *p* < .001, η_p_^2^ = 0.719], and no significant Arrangement by Emotion interaction [*F*(2, 126) = 2.119, *p* = .124, η_p_^2^ = 0.033] (Fig. 3C).

For the significant main effect of Emotion, individuals presented within Happy pairs (*M* = 21.51, *SD* = 12.31) were rated as significantly less negative than Angry (*M* = 55.73, *SD* = 13.22) pairs [*t*(63) = 20.698, *p* < .001, *d* = 2.587, BF_01_ < 0.001], and Neutral (*M* = 39.55, *SD* = 17.92) pairs [*t*(63) = 8.461, *p* < .001, *d* = 1.058, BF_01_ < 0.001]. Individuals presented within Neutral pairs were perceived as less negative than those presented within Angry pairs [*t*(63) = 8.512, *p* < .001, *d* = 1.064, BF_01_ < 0.001].

## General Discussion

In Experiment 1, happy dyads were rated as more positive and less negative when they were presented face-to-face than back-to-back, and angry dyads were rated as more negative when presented face-to-face than back-to-back. In Experiment 2, when presented for 500ms, valence intensity ratings of angry face-to-face dyads were more negative and less positive than the same stimuli presented back-to-back. The effects on valence intensity were consistent with the effects found for the categorisation of emotion in dyads (Abramson et al., 2021; Gray et al., 2017), such that the perception of intensity was increased consistent with the affect that was displayed (e.g., angry was perceived as more negative, and happy as more positive).

To investigate whether the effect of arrangement on perceived valence intensity was driven by attentional cues, we conducted Experiment 3, in which one of the individuals in the dyad was replaced by an arrow. Recent research suggests that attentional cueing is an important factor in the perception of interacting dyads (Vestner et al., 2020; Vestner, Gray et al., 2021; Vestner, Gray et al., 2022; Vestner, Over et al., 2022). Using a visual search task, Vestner et al. (2020) found dyad prioritisation effects for face-to-face bodies, point-to-point arrows, and face-to-point mixed displays (versus back-to-back bodies, base-to-base arrows, and back-to-base mixed displays, respectively), suggesting that the attentional capture of face-to-face bodies is not specific to social interactions, but is driven by their attentional cueing properties. Enhanced visual search has also been found for facing versus non-facing objects, but only for those objects that also cued spatial attention, such as lamps and cameras (Vestner, Gray et al., 2022; Vestner, Over et al., 2022). As facing dyads capture attention, we reasoned that increased perceived valence intensity for facing dyads might be driven by increased attentional processing, in a similar way to the effects of attention on low-level vision (e.g., Pestilli & Carrasco, 2005). However, we found that an individual was not rated as more intensely emotional when presented face-to-point than back-to-base when an attentionally-directing arrow stimulus replaced one of the individuals in the dyad. Therefore, attentional cueing is unlikely to drive the effects reported here.

With unlimited time to view the stimuli, we found dyad arrangement affected valence intensity perception for both happy and angry expressions. Previous research has not consistently found effects of angry context interactants on the emotion categorisation of a target expression (Abramson et al., 2021; Gray et al., 2017). For example, Abramson et al. (2021) did not find a difference in categorisation accuracy of an angry target when presented alongside context interactants that were angry or fearful. This highlights an advantage of the current study, where the measurement of emotional intensity allowed us to investigate effects that might not be detectable using emotion categorisation tasks.

Dyad arrangement affected the perceived intensity of angry bodies when they were presented for a short duration, and both angry and happy bodies when they were presented for longer. It is possible that the impact of interaction arrangement on happy expressions is the product of more careful examination of the stimuli, whereas for angry expressions, the effect is more rapidly accrued. Evidence suggests that angry expressions are processed more efficiently than other, particularly non-threat-related, expressions (Calvo et al., 2006; Maratos et al., 2008), and our results are consistent with this view. An alternative explanation is that, as the angry expressions were more ambiguous than happy expressions (i.e., they were rated as less negative than happy expressions were rated as positive), the angry expressions were influenced by context more easily. However, as only one positive and one negative expression were used in the current experiment, the specificity of the effect to angry, negative, or ambiguous expressions is unclear. Future experiments should investigate the perception of briefly presented dyads using a greater number of emotions to ascertain whether this is valence or emotion specific. They could also incorporate response time measurements in combination with drift diffusion modelling to provide further insights into the perceptual/decision making processes.

Some have argued that the perception of social interactions recruit specialised processing (Papeo, 2020). The results of the current studies suggest that emotional social interactions are processed differently to non-interactions. It is possible that the effect is driven by configural processing, which is thought to provide greater sensitivity to the spatial relationships between features for upright than inverted faces (Farah et al., 1998; Maurer et al., 2002; Piepers & Robbins, 2012), and has been argued to be responsible for some of the effects found within social interaction research (Abassi & Papeo, 2022; Papeo, 2020; Papeo et al., 2017). However, recent research suggests that facing dyads are not more likely to be processed configurally than non-facing dyads (Bunce et al., 2024). An alternative hypothesis is that detection of one interactant suggests perceptual hypotheses about the other, derived from internal models of social interactions (Friston, 2005; Gregory, 1997). Exposure to the emotional contingencies viewed in everyday life are likely to exert an influence on our internal models of social interactions.

In our study, we manipulated dyad arrangement by presenting stimuli face-to-face and back-to-back. This manipulation has been used widely in the literature (e.g., Abassi & Papeo, 2020; Bunce et al., 2021; Gandolfo et al., 2024; Gray et al., 2017; Papeo et al., 2017; Vestner et al., 2019). However, to interpret the more unusual back-to-back presentations, observers may construct explanations for them. For example, happy back-to-back dyads might depict the celebration of a victor in a competitive situation, whereas angry back-to-back dyads might depict a social scene immediately before or immediately after an aggressive confrontation. Further research could explore participants’ interpretation of back-to-back interactions.

In conclusion, happy and angry facing dyads were rated as more positive and negative, respectively, than non-facing dyads, suggesting that groups of individuals interacting are interpreted as more intensely emotional than the same individuals not interacting. The effect for angry facing versus non-facing dyads was found even when the stimuli were presented briefly. Importantly, we found that these results were unlikely to be driven by directional attention cues.

## Data Availability

A link to the data is provided in the manuscript.

## References

[CR1] Abassi, E., & Papeo, L. (2020). The representation of two-body shapes in the human visual cortex. *The Journal of Neuroscience,**40*(4), 852. 10.1523/JNEUROSCI.1378-19.2019 31801812 10.1523/JNEUROSCI.1378-19.2019 PMC6975292

[CR2] Abramson, L., Petranker, R., Marom, I., & Aviezer, H. (2021). Social interaction context shapes emotion recognition through body language, not facial expressions. *Emotion,**21*(3), 557–568. 10.1037/emo0000718 31971411 10.1037/emo0000718

[CR3] Adams, R. B., Jr., & Kleck, R. E. (2005). Effects of direct and averted gaze on the perception of facially communicated emotion. *Emotion,**5*(1), 3–11. 10.1037/1528-3542.5.1.3 15755215 10.1037/1528-3542.5.1.3

[CR4] Adolphs, R. (2002). Recognizing emotion from facial expressions: Psychological and neurological mechanisms. *Behavioral and Cognitive Neuroscience Reviews,**1*(1), 21–62. 10.1177/1534582302001001003 17715585 10.1177/1534582302001001003

[CR5] Anwyl-Irvine, A. L., Massonnié, J., Flitton, A., Kirkham, N., & Evershed, J. K. (2020). Gorilla in our midst: An online behavioral experiment builder. *Behavior Research Methods,**52*(1), 388–407. 10.3758/s13428-019-01237-x 31016684 10.3758/s13428-019-01237-x PMC7005094

[CR6] Atkinson, A. P., Dittrich, W. H., Gemmell, A. J., & Young, A. W. (2004). Emotion perception from dynamic and static body expressions in point-light and full-light displays. *Perception,**33*(6), 717–746. 10.1068/p5096 15330366 10.1068/p5096

[CR7] Aviezer, H., Hassin, R. R., Ryan, J., Grady, C., Susskind, J., Anderson, A., Moscovitch, M., & Bentin, S. (2008). Angry, disgusted, or afraid?: Studies on the malleability of emotion perception. *Psychological Science,**19*(7), 724–732. 10.1111/j.1467-9280.2008.02148.x 18727789 10.1111/j.1467-9280.2008.02148.x

[CR8] Aviezer, H., Trope, Y., & Todorov, A. (2012). Holistic person processing: Faces with bodies tell the whole story. *Journal of Personality and Social Psychology,**103*(1), 20–37. 10.1037/a0027411 22352325 10.1037/a0027411

[CR9] Bachmann, J., Krüger, B., Keck, J., Munzert, J., & Zabicki, A. (2022). When the timing is right: The link between temporal coupling in dyadic interactions and emotion recognition. *Cognition,**229*, Article 105267. 10.1016/j.cognition.2022.105267 36058018 10.1016/j.cognition.2022.105267

[CR10] Barrett, L. F., Mesquita, B., & Gendron, M. (2011). Context in emotion perception. *Current Directions in Psychological Science,**20*(5), 286–290. 10.1177/0963721411422522

[CR11] Barzy, M., Morgan, R., Cook, R., & Gray, K. L. (2023). Are social interactions preferentially attended in real-world scenes? Evidence from change blindness. *Quarterly Journal of Experimental Psychology*, 17470218231161044. 10.1177/17470218231161044 10.1177/17470218231161044 PMC10503233 36847458

[CR12] Batty, M., & Taylor, M. J. (2003). Early processing of the six basic facial emotional expressions. *Cognitive Brain Research,**17*(3), 613–620. 10.1016/S0926-6410(03)00174-5 14561449 10.1016/s0926-6410(03)00174-5

[CR13] Bunce, C., Gray, K. L. H., & Cook, R. (2021). The perception of interpersonal distance is distorted by the Müller-Lyer illusion. *Scientific Reports,**11*(1), 494. 10.1038/s41598-020-80073-y 33436801 10.1038/s41598-020-80073-y PMC7803751

[CR14] Calvo, M. G., Avero, P., & Lundqvist, D. (2006). Facilitated detection of angry faces: Initial orienting and processing efficiency. *Cognition and Emotion,**20*(6), 785–811. 10.1080/02699930500465224

[CR15] Clarke, T. J., Bradshaw, M. F., Field, D. T., Hampson, S. E., & Rose, D. (2005). The perception of emotion from body movement in point-light displays of interpersonal dialogue. *Perception,**34*(10), 1171–1180. 10.1068/p5203 16309112 10.1068/p5203

[CR16] Cline, M. G. (1956). The influence of social context on the perception of faces. *Journal of Personality,**25*(2), 142–158. 10.1111/j.1467-6494.1956.tb01294.x 13385788 10.1111/j.1467-6494.1956.tb01294.x

[CR17] Crump, M. J. C., McDonnell, J. V., & Gureckis, T. M. (2013). Evaluating amazon’s mechanical turk as a tool for experimental behavioral research. *PLoS ONE,**8*(3), Article e57410. 10.1371/journal.pone.0057410 23516406 10.1371/journal.pone.0057410 PMC3596391

[CR18] de Gelder, B. (2006). Towards the neurobiology of emotional body language. *Nature Reviews Neuroscience,**7*(3), 242–249. 10.1038/nrn1872 16495945 10.1038/nrn1872

[CR19] de Gelder, B., & Vroomen, J. (2000). The perception of emotions by ear and by eye. *Cognition and Emotion,**14*(3), 289–311. 10.1080/026999300378824

[CR20] Farah, M. J., Wilson, K. D., Drain, M., & Tanaka, J. N. (1998). What is ‘special’ about face perception? *Psychological Review,**105*(3), 482–498. 10.1037/0033-295X.105.3.482 9697428 10.1037/0033-295x.105.3.482

[CR21] Faul, F., Erdfelder, E., Lang, A.-G., & Buchner, A. (2007). G*Power 3: A flexible statistical power analysis program for the social, behavioral, and biomedical sciences. *Behavior Research Methods,**39*(2), 175–191. 10.3758/BF03193146 17695343 10.3758/bf03193146

[CR22] Friston, K. (2005). A theory of cortical responses. *Philosophical Transactions of the Royal Society b: Biological Sciences,**360*(1456), 815–836. 10.1098/rstb.2005.1622 10.1098/rstb.2005.1622 PMC1569488 15937014

[CR23] Gandolfo, M., Abassi, E., Balgova, E., Downing, P. E., Papeo, L., & Koldewyn, K. (2024). Converging evidence that left extrastriate body area supports visual sensitivity to social interactions. *Current Biology,**34*(2), 343-351.e5. 10.1016/j.cub.2023.12.009 38181794 10.1016/j.cub.2023.12.009

[CR24] Gelder, B. de, Meeren, H. K. M., Righart, R., Stock, J. van den, van de Riet, W. A. C., & Tamietto, M. (2006). Beyond the face: Exploring rapid influences of context on face processing. In S. Martinez-Conde, S. L. Macknik, L. M. Martinez, J.-M. Alonso, & P. U. Tse (Eds.), *Progress in Brain Research* (Vol. 155, pp. 37–48). Elsevier. 10.1016/S0079-6123(06)55003-4 10.1016/S0079-6123(06)55003-4 17027378

[CR25] Germine, L., Nakayama, K., Duchaine, B. C., Chabris, C. F., Chatterjee, G., & Wilmer, J. B. (2012). Is the Web as good as the lab? Comparable performance from Web and lab in cognitive/perceptual experiments. *Psychonomic Bulletin & Review,**19*(5), 847–857. 10.3758/s13423-012-0296-9 22829343 10.3758/s13423-012-0296-9

[CR26] Goupil, N., Rayson, H., Serraille, É., Massera, A., Ferrari, P. F., Hochmann, J.-R., & Papeo, L. (2024). Visual preference for socially relevant spatial relations in humans and monkeys. *Psychological Science*, 09567976241242995. 10.1177/09567976241242995 10.1177/09567976241242995 38683657

[CR27] Gray, K. L. H., Barber, L., Murphy, J., & Cook, R. (2017). Social interaction contexts bias the perceived expressions of interactants. *Emotion,**17*(4), 567–571. 10.1037/emo0000257 28191995 10.1037/emo0000257

[CR28] Gray, K. L. H., Flack, T. R., Yu, M., Lygo, F. A., & Baker, D. H. (2020). Nonlinear transduction of emotional facial expression. *Vision Research,**170*, 1–11. 10.1016/j.visres.2020.03.004 32217366 10.1016/j.visres.2020.03.004

[CR29] Gregory, R. L. (1997). Knowledge in perception and illusion. *Philosophical Transactions of the Royal Society of London. Series B, Biological Sciences*, *352*(1358), 1121–1127. PubMed. 10.1098/rstb.1997.0095 10.1098/rstb.1997.0095 PMC1692018 9304679

[CR30] Hess, U., Blairy, S., & Kleck, R. E. (1997). The intensity of emotional facial expressions and decoding accuracy. *Journal of Nonverbal Behavior,**21*(4), 241–257. 10.1023/A:1024952730333

[CR31] Heyes, C. (2014). False belief in infancy: A fresh look. *Developmental Science,**17*(5), 647–659. 10.1111/desc.12148 24666559 10.1111/desc.12148

[CR32] Isik, L., Koldewyn, K., Beeler, D., & Kanwisher, N. (2017). Perceiving social interactions in the posterior superior temporal sulcus. *Proceedings of the National Academy of Sciences,**114*(43), E9145–E9152. 10.1073/pnas.1714471114 10.1073/pnas.1714471114 PMC5664556 29073111

[CR33] van Kleef, G. A., Cheshin, A., Fischer, A. H., & Schneider, I. K. (2016). Editorial: The social nature of emotions. *Frontiers in Psychology*, *7*. https://www.frontiersin.org/articles/10.3389/fpsyg.2016.00896 10.3389/fpsyg.2016.00896 PMC4906017 27378990

[CR34] Kret, M. E., & de Gelder, B. (2010). Social context influences recognition of bodily expressions. *Experimental Brain Research,**203*(1), 169–180. 10.1007/s00221-010-2220-8 20401473 10.1007/s00221-010-2220-8 PMC2862946

[CR35] Kuhn, G., & Kingstone, A. (2009). Look away! Eyes and arrows engage oculomotor responses automatically. *Attention, Perception, & Psychophysics,**71*(2), 314–327. 10.3758/APP.71.2.314 10.3758/APP.71.2.314 19304621

[CR36] Leppänen, J. M., Kauppinen, P., Peltola, M. J., & Hietanen, J. K. (2007). Differential electrocortical responses to increasing intensities of fearful and happy emotional expressions. *Brain Research,**1166*, 103–109. 10.1016/j.brainres.2007.06.060 17662698 10.1016/j.brainres.2007.06.060

[CR37] Liu, T., Abrams, J., & Carrasco, M. (2009). Voluntary Attention Enhances Contrast Appearance. *Psychological Science,**20*(3), 354–362. 10.1111/j.1467-9280.2009.02300.x 19254239 10.1111/j.1467-9280.2009.02300.x PMC2657200

[CR38] Maratos, F. A., Mogg, K., & Bradley, B. P. (2008). Identification of angry faces in the attentional blink. *Cognition and Emotion,**22*(7), 1340–1352. 10.1080/02699930701774218 19360116 10.1080/02699930701774218 PMC2666369

[CR39] Marsh, A. A., Ambady, N., & Kleck, R. E. (2005). The Effects of Fear and Anger Facial Expressions on Approach- and Avoidance-Related Behaviors. *Emotion,**5*(1), 119–124. 10.1037/1528-3542.5.1.119 15755225 10.1037/1528-3542.5.1.119

[CR40] Masuda, T., Ellsworth, P. C., Mesquita, B., Leu, J., Tanida, S., & Van de Veerdonk, E. (2008). Placing the face in context: Cultural differences in the perception of facial emotion. *Journal of Personality and Social Psychology,**94*(3), 365–381. 10.1037/0022-3514.94.3.365 18284287 10.1037/0022-3514.94.3.365

[CR41] Maurer, D., Le Grand, R., & Mondloch, C. J. (2002). The many faces of configural processing. *Trends in Cognitive Sciences,**6*(6), 255–260. 10.1016/S1364-6613(02)01903-4 12039607 10.1016/s1364-6613(02)01903-4

[CR42] Meeren, H. K. M., van Heijnsbergen, C. C. R. J., & de Gelder, B. (2005). Rapid perceptual integration of facial expression and emotional body language. *Proceedings of the National Academy of Sciences,**102*(45), 16518–16523. 10.1073/pnas.0507650102 10.1073/pnas.0507650102 PMC1283446 16260734

[CR43] Mumenthaler, C., & Sander, D. (2012). Social appraisal influences recognition of emotions. *J Pers Soc Psychol*, *102*(6), 1118–1135. PubMed. 10.1037/a0026885 10.1037/a0026885 22288528

[CR44] Mumenthaler, C., & Sander, D. (2015). Automatic integration of social information in emotion recognition. *Journal of Experimental Psychology: General,**144*(2), 392. 25688908 10.1037/xge0000059

[CR45] Öhman, A. (2002). Automaticity and the Amygdala: Nonconscious Responses to Emotional Faces. *Current Directions in Psychological Science,**11*(2), 62–66. 10.1111/1467-8721.00169

[CR46] Papeo, L. (2020). Twos in human visual perception. *Cortex,**132*, 473–478. 10.1016/j.cortex.2020.06.005 32698947 10.1016/j.cortex.2020.06.005

[CR47] Papeo, L., Stein, T., & Soto-Faraco, S. (2017). The two-body inversion effect. *Psychological Science,**28*(3), 369–379. 10.1177/0956797616685769 28140764 10.1177/0956797616685769

[CR48] Papeo, L., Goupil, N., & Soto-Faraco, S. (2019). Visual search for people among people. *Psychological Science,**30*(10), 1483–1496. 10.1177/0956797619867295 31532709 10.1177/0956797619867295

[CR49] Pelz, J. B., & Canosa, R. (2001). Oculomotor behavior and perceptual strategies in complex tasks. *Vision Research,**41*(25), 3587–3596. 10.1016/S0042-6989(01)00245-0 11718797 10.1016/s0042-6989(01)00245-0

[CR50] Pestilli, F., & Carrasco, M. (2005). Attention enhances contrast sensitivity at cued and impairs it at uncued locations. *Vision Research,**45*(14), 1867–1875. 10.1016/j.visres.2005.01.019 15797776 10.1016/j.visres.2005.01.019

[CR51] Piepers, D. W., & Robbins, R. A. (2012). A review and clarification of the terms “holistic,” “configural,” and “relational” in the face perception literature. *Frontiers in Psychology*, *3*. 10.3389/fpsyg.2012.00559 10.3389/fpsyg.2012.00559 PMC3571734 23413184

[CR52] Quadflieg, S., & Koldewyn, K. (2017). The neuroscience of people watching: How the human brain makes sense of other people’s encounters. *Annals of the New York Academy of Sciences,**1396*(1), 166–182. 10.1111/nyas.13331 28405964 10.1111/nyas.13331

[CR53] Reschke, P. J., & Walle, E. A. (2021). The unique and interactive effects of faces, postures, and scenes on emotion categorization. *Affective Science,**2*(4), 468–483. 10.1007/s42761-021-00061-x 36046211 10.1007/s42761-021-00061-x PMC9382938

[CR54] Righart, R., & de Gelder, B. (2008). Rapid influence of emotional scenes on encoding of facial expressions: An ERP study. *Social Cognitive and Affective Neuroscience,**3*(3), 270–278. 10.1093/scan/nsn021 19015119 10.1093/scan/nsn021 PMC2566764

[CR55] Santiesteban, I., Catmur, C., Hopkins, S. C., Bird, G., & Heyes, C. (2014). Avatars and arrows: Implicit mentalizing or domain-general processing? *Journal of Experimental Psychology: Human Perception and Performance,**40*(3), 929–937. 10.1037/a0035175 24377486 10.1037/a0035175

[CR56] Thoma, P., Soria Bauser, D., & Suchan, B. (2013). BESST (Bochum Emotional Stimulus Set)—A pilot validation study of a stimulus set containing emotional bodies and faces from frontal and averted views. *Psychiatry Research,**209*(1), 98–109. 10.1016/j.psychres.2012.11.012 23219103 10.1016/j.psychres.2012.11.012

[CR57] Tipples, J. (2002). Eye gaze is not unique: Automatic orienting in response to uninformative arrows. *Psychonomic Bulletin & Review,**9*(2), 314–318. 10.3758/BF03196287 12120794 10.3758/bf03196287

[CR58] Tsantani, M., Podgajecka, V., Gray, K. L. H., & Cook, R. (2022). How does the presence of a surgical face mask impair the perceived intensity of facial emotions? *PLoS ONE,**17*(1), Article e0262344. 10.1371/journal.pone.0262344 35025948 10.1371/journal.pone.0262344 PMC8758043

[CR59] Vestner, T., Tipper, S. P., Hartley, T., Over, H., & Rueschemeyer, S.-A. (2019). Bound together: Social binding leads to faster processing, spatial distortion, and enhanced memory of interacting partners. *Journal of Experimental Psychology: General,**148*(7), 1251–1268. 10.1037/xge0000545 30652892 10.1037/xge0000545

[CR60] Vestner, T., Gray, K. L. H., & Cook, R. (2020). Why are social interactions found quickly in visual search tasks? *Cognition,**200*, Article 104270. 10.1016/j.cognition.2020.104270 32220782 10.1016/j.cognition.2020.104270 PMC7315127

[CR61] Vestner, T., Gray, K. L. H., & Cook, R. (2021). Visual search for facing and non-facing people: The effect of actor inversion. *Cognition,**208*, 10.1016/j.cognition.2020.104550 33360076

[CR62] Vestner, T., Over, H., Gray, K.L.H., Tipper, S.T., & Cook, R. (2021). Searching for people: Non-facing distractor pairs hinder the visual search of social scenes more than facing distractor pairs. *Cognition, 214*, 104737. 10.1016/j.cognition.2021.104737 10.1016/j.cognition.2021.104737 PMC8346951 33901835

[CR63] Vestner, T., Gray, K. L. H., & Cook, R. (2022). Sensitivity to orientation is not unique to social attention cueing. *Scientific Reports,**12*(1), 5059. 10.1038/s41598-022-09011-4 35322128 10.1038/s41598-022-09011-4 PMC8943057

[CR64] Vestner, T., Over, H., Gray, K. L. H., & Cook, R. (2022). Objects that direct visuospatial attention produce the search advantage for facing dyads. *Journal of Experimental Psychology: General,**151*(1), 161–171. 10.1037/xge0001067 34110891 10.1037/xge0001067

[CR65] Wronka, E., & Walentowska, W. (2011). Attention modulates emotional expression processing. *Psychophysiology,**48*(8), 1047–1056. 10.1111/j.1469-8986.2011.01180.x 21332489 10.1111/j.1469-8986.2011.01180.x

